# Regulation of long noncoding RNAs in the pathogenesis and clinical implications of pituitary adenomas

**DOI:** 10.1002/iid3.1047

**Published:** 2023-10-13

**Authors:** Ling Wang, Dingkai Xu

**Affiliations:** ^1^ Department of Endocrinology Liangzhou Hospital Wuwei Gansu China; ^2^ Department of Neurosurgery Liangzhou Hospital Wuwei Gansu China

**Keywords:** biomarker, ceRNAs, diagnosis, LncRNAs, pituitary adenoma

## Abstract

**Background:**

Pituitary adenoma (PA) is a type of tumor that develops in the sella turcica and is one of the most frequent intracranial tumors. It belongs to a type of adenoma derived from a single clone of cells in the pituitary gland. PA ranks third among all intracranial tumors, following only gliomas and meningioma. The average prevalence rate is approximately 15% at autopsy and 22.5% at radiological examinations.

**Objective and Significance:**

Most PAs are benign and non‐invasive adenomas that can be removed surgically or controlled with medication. However, approximately 35% of them show invasion into nearby anatomical structures and cannot be completely resected. 0.1%~0.2% of PA cases eventually develop into pituitary carcinomas. Additionally, PA may cause severe morbidity due to mass effects and the disorder of pituitary hormone secretion. Therefore, there is an urgent need to clarify the pathological mechanism of PA, improve the accuracy of diagnosis, and develop targeted therapies.

**Research Status:**

Although current knowledge about the pathogenesis of PA remains limited, epigenetic modulation of PA has been increasingly implicated. Long non‐coding RNAs (lncRNAs) are known to regulate gene expression post‐transcriptionally and exert substantial roles in the initiation, progression, or suppression of various tumors. Accumulating evidence has shown close relationships between lncRNA dysregulation and PA development.

**Conclusions:**

This review highlights recent progress in the study of lncRNAs in PA pathogenesis and their potential as diagnostic/prognostic biomarkers or therapeutic targets for PA patients.

## 1 INTRODUCTION

As one of the most frequent intracranial neoplasms, pituitary adenoma (PA) accounts for approximately 10%−15% of all surgically resected intracranial tumors, with a reported prevalence of 1 in 1000 of the general population.^1^, ^2^ PA is usually a benign lesion, but some PA patients show aggressive characteristics, such as local invasion, rapid growth, and poor responses to conventional therapies.^2^ PA can be classified into functional PA (FPA) and nonfunctional PA (NFPA),^3^ or divided into invasive PA (IPA) and noninvasive PA (NIPA).^4^ IPA displays high proliferative and invasive properties, tending to invade surrounding structures, including the cavernous sinuses, cranial nerves, and nearby vascular structures.^5^, ^6^ Due to its aggressive behavior, IPA cannot be completely resected through surgery, leading to a low rate of recovery and a high incidence of recurrence in patients.^7^ Therefore, the identification of novel biomarkers for early accurate detection and prognostic evaluation of IPA is of significant importance. In addition, the exploration of molecular mechanisms underlying PA pathogenesis to identify the optimal therapeutic targets is becoming increasingly more relevant.

Long noncoding RNAs (lncRNAs) belong to a class of evolutionarily conserved and endogenously expressed noncoding RNAs. They are over 200 nucleotides in length and have epigenetic regulatory potential.^8^ Over the past decades, numerous studies have been conducted to reveal lncRNA function in human cells, such as modulating gene activation and repression, chromosome modification, X chromosome inactivation, transcriptional interference, posttranslational regulation, and alternative splicing.^9^, ^10^ LncRNAs perform the above functions via diverse mechanisms. For example, they act as competing endogenous RNAs (ceRNAs) that sequester microRNAs (miRNAs) and thus terminate their suppression of target genes,^11^ enhance or impair long‐range chromatin interactions,^12^ and act as molecular scaffolds that bring together chromatin and histone‐modifying complexes.^13^ Given the importance of lncRNAs in modulating epigenetic processes, the dysregulation of lncRNAs has been implicated in a variety of human diseases, including tumor.^14^ Moreover, increasing evidence has confirmed the participation of lncRNAs in the proliferation, invasion, migration, glycolysis, and epithelial−mesenchymal transformation (EMT) during PA development.^8^, ^15^ Here we highlight recent findings concerning lncRNAs and their relevance to the pathogenesis of PA. The aim is to provide a theoretical basis for developing diagnostic and prognostic tools with high specificity, classification criteria with high accuracy, and novel therapeutic strategies with high potency in the future.

## 2 CURRENT RESEARCH ON PA

**Figure 1:**
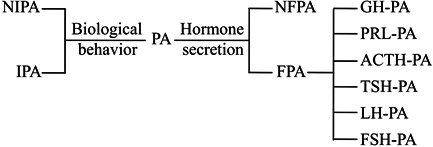
The classification of pituitary adenomas. ACTH, adrenocorticotropic hormone; FPA, functional pituitary adenomas; FSH, follicle‐stimulating hormone; GH, growth hormone; IPA, invasive pituitary adenomas; LH, luteinizing hormone; NFPA, nonfunctional pituitary adenomas; NIPA, noninvasive pituitary adenomas; PA, pituitary adenomas; PRL, prolactinoma; TSH, thyroid‐stimulating hormone.

PA is a type of benign epithelial tumor that arises from the anterior pituitary gland. It can be classified according to different criteria.^16^ For example, based on size, PA can be divided into microadenoma with a diameter of less than 1 cm, large adenoma with a diameter between 1 and 4 cm, and giant adenoma with a diameter of more than 4 cm. According to imaging features and biological behaviors, PA can be classified as IPA and NIPA. The invasiveness of PA is usually assessed by the Knosp Grading System based on its relationship with internal carotid arteries.^17^ Based on the capability of hormone secretion, PA is sorted into NFPA and FPA; the latter further includes growth hormone (GH) adenoma, prolactin (PRL)‐secreting adenomas (also known as prolactinoma), adrenocorticotropic hormone (ACTH) adenoma, thyroid‐stimulating hormone (TSH) adenoma, luteinizing hormone (LH) adenoma, and follicle‐stimulating hormone (FSH) adenoma (see Figure 1).^18^ Among these, prolactinoma and NFPA, accounting for about 40% and 20%, respectively, are the two most predominant subtypes of PA.^19^

Currently, several therapeutic options are available for PA, including surgery, radiotherapy, chemotherapy, immunotherapy, as well as the emerging molecular‐targeted therapy.^20^, ^21^ Dopamine agonists, including bromocriptine and cabergoline, are the first‐line treatment for prolactinoma by suppressing prolactin hypersecretion, reducing tumor size, and restoring gonadal function.^22^ Surgical resection via endoscopic/microscopic operation or craniotomy is the primary choice for other PA types excluding prolactinoma.^23^ A recent meta‐analysis comparing endoscopic endonasal and microscopic transsphenoidal surgery for relapsing/residual PA has shown that the endoscopic approach moderately increased the rate of gross‐total tumor resection and improved endocrine and visual functions.^24^ Another multicentric retrospective study has demonstrated that the transsphenoidal resection of adenoma was still the first choice in the majority of pediatric PA patients, with relief of mass effect and recovery of normal endocrine function.^25^ However, these procedures are not effective for a considerable portion of PA patients, making it a challenge in the clinic. The continuous discovery of novel lncRNAs and clarification of their biological functions provide us with new insights into the specific pathogenesis of PA, and unfold a broad prospect for the study on the diagnosis, treatment, and prognosis of PA.^26^, ^27^

## 3 LNCRNA‐INVOLVED MECHANISMS IN PA PATHOLOGY

### 3.1 The characteristics of lncRNAs

LncRNAs represent a class of RNA transcripts with lengths over 200 nucleotides. They display higher spatial and temporal specificity and lower conservation compared to mRNAs.^8^ Previously regarded as transcriptional by‐products, lncRNAs have now been widely recognized as active modulators in processes related to cell growth, differentiation, apoptosis, and autophagy, and are also involved in the pathogenesis of diverse diseases such as brain tumor and cancer.^28^ According to their location in the genome relative to the target protein‐coding genes, lncRNAs are divided into sense, antisense, bidirectional, intronic, intergenic, and enhancer‐associated lncRNAs.^29^ LncRNAs regulate gene expression at epigenetic, transcriptional, and posttranscriptional levels.^30^ For instance, miRNAs are known to cause mRNA cleavage or translational repression of target genes by complementary pairing with the 3′‐untranslated region of mRNAs, modulating gene expression at the posttranscriptional level. MiRNA sponges are ceRNAs that can indirectly influence target gene expression through competitive binding to miRNAs.^11^ A lot of lncRNAs have been found to modulate gene expression and tumor progression by acting as miRNA sponges.^31^, ^32^

Recently, more and more evidence has described the aberrant expression of multiple lncRNAs being involved in the occurrence and progression of PA. For example, lncRNA CCAT2 was dramatically induced in PA tissues and correlated with the poor prognosis in PA patients. Further loss‐of‐function and gain‐of‐function experiments found that CCAT2 positively modulated the proliferation, migration, and invasion of PA cells, and displayed an oncogenic property in PA carcinogenesis and progression.^33^ Another study showed that the expression of lncRNA H19 was frequently decreased in human primary PA and negatively related to tumor development.^34^ A genome‐wide analysis of lncRNAs by RNA‐seq identified 839 differentially expressed lncRNAs in pituitary tissues from patients with gonadotrophin‐secreting PA and normal people.^35^

### 3.2 Regulation of lncRNAs in PA proliferation and apoptosis

IPA is characterized by exuberant cell proliferation, which is an important indicator for measuring the invasiveness of PA.^4^ Some lncRNAs have been found to affect cell cycle and thus modulate the proliferation of PA cells by direct or indirect regulation of cyclin expression.^36^ For instance, the expression of lncRNA MEG3 was significantly lower in PA in compared with adjacent normal tissues, as detected by RT‐qPCR. Overexpression of lncRNA MEG3 inhibited proliferation and accelerated apoptosis of PA cells.^37^ LncRNA SNHG7 showed an aberrant upregulation in both PA tissues and cell lines, and was associated with poor survival outcome. Knockdown of SNHG7 suppressed cell proliferation and promoted apoptosis.^38^ LncRNA RPSAP52 was highly expressed in gonadotroph and PRL PAs, where it positively correlated to the expression of HMGA proteins. Functional study revealed that RPSAP52 promoted cell proliferation by accelerating the transition from G1 phase to S phase during the cell cycle.^39^ LINC00473 was overexpressed in IPA, it increased the expression of cyclin D1 and CDK2, two markers for the cell cycle process, and thus promoted the proliferation of PA cells.^40^ LncRNA AFAP1‐AS1 was shown to bind to miR‐103a‐3p in rat PA cells, while miR‐103a‐3p overexpression could suppress rat PA cell proliferation, induce apoptosis, arrest cell cycle in the G/S phase, reduce GH and PRL secretion, and inhibit the PI3K/AKT signaling pathway.^41^

### 3.3 Regulation of lncRNAs in PA migration and invasion

IPA cells possess high aggressive characteristics and can invade and damage adjacent tissues, including the sellar area and cavernous sinus.^42^ A majority of lncRNAs have been validated to function as proto‐oncogenes and can directly promote tumor cell migration and invasion and participate in PA progression. For instance, the lncRNA MEG3 level was found to be lower in patients with stage III−IV NFPA than those with stage I−II, with the former displaying a higher invasiveness than the latter, while overexpression of MEG3 inhibited the migration and invasion of PA tumor cells.^37^ LncRNA MYMLR was obviously upregulated in PA tissues compared to that in normal controls.^43^ MYMLR modulated cell migration and invasion by sponging miR‐197‐3p and counteracted miR‐197‐3p's tumor suppressive effect by negatively regulating carbonyl reductase 1 (CBR1).^43^ LncRNA TUG1 was observed to be upregulated in PA tissues and related with tumor invasion, knosp grade, and tumor size. TUG1 knockdown inhibited cell invasion and migration by modulating NF‐κB signaling pathway.^44^ The expression of lncRNA BBOX 1‐AS1 increased in PA tissues and cells, its downregulation causing the inhibition of PA invasion and progression in vivo,^45^ opening up a new path for the treatment of PA.

### 3.4 Regulation of lncRNAs in EMT progress

EMT is a process whereby polarized epithelial cells are transformed into mesenchymal cells and display attenuated cell adhesion, enhanced motility, and invasive properties.^4^, ^46^ Studies have found that the mesenchymal markers in IPA were overexpressed, while the epithelial markers were downregulated.^47^ The invasion and metastasis of IPA may be related to EMT, and lncRNAs might participate in PA pathogenesis by regulating the EMT process. For instance, levels of EMT markers were regulated after MEG3 overexpression: E‐cadherin was upregulated, while Twist, Slug and MMP‐7 were downregulated, suggesting that MEG3 suppressed pituitary tumor development by participating in the EMT process.^37^ LncRNA ST8SIA6‐AS1 was highly expressed in IPA, the downregulation of ST8SIA6‐AS1 by gene interference increased E‐cadherin expression while decreased the expression of vimentin, N‐cadherin, and several tumor‐associated transcription factors, indicating an inhibition of the EMT process by ST8SIA6‐AS1 knockdown.^48^ Therefore, as an important mechanism underlying PA pathogenesis, reversing, or blocking the EMT process by regulating specific lncRNAs or serving as therapeutic targe might provide a novel strategy to interfere with PA development.

### 3.5 Regulation of lncRNAs in PA glycolysis

IPA cells are inclined to undergo glycolysis preferentially to adapt to changes in the surrounding environment under aerobic conditions, which is known as the “Warburg effect.”^49^ The involvement of lncRNAs is essential for the glycolysis of tumor cells.^50^ Many lncRNAs have been found to directly modulate the expression of glycolytic enzymes or regulate glycolysis‐related oncogenes, however, there is still a lack of relevant reports between lncRNAs and PA. A study by proteomics and metabolomics revealed significant differences in proteins and metabolites between ACTH‐PA and normal pituitary glands.^51^ Further protein−metabolite interaction analysis showed that these differentially expressed proteins and metabolites were predominantly enriched in glycolysis in ACTH‐PA.^51^ LncRNA‐UCA1 was highly expressed in PA, and significantly upregulated HK2 and LDHA, two key glycolytic enzymes involved in glycolysis.^52^ Meanwhile, the glycolysis in PA tissues was higher than that in normal pituitary tissues.^52^ Overexpression of UCA1 in rat PA cell lines significantly enhanced glucose uptake and lactate secretion, while lncRNA‐UCA1 knockdown suppressed PA cell growth and prolactin production.^52^ These findings revealed an oncogenic role of UCA1 by upregulating glycolysis in PA and contributed to the understanding of the underlying mechanisms of PA tumorigenesis.

## 4 THE CLINICAL IMPLICATION OF LNCRNAS IN PA

### 4.1 LncRNAs as biomakers for PA

#### 4.1.1 Diagnostic biomarkers

The major difficulty in defining a PA as aggressive is the absence of predictive biomarkers. A recent microarray analysis revealed the expression patterns of lncRNAs in IPA, and identified 81 upregulated lncRNAs and 165 downregulated lncRNAs in IPA compared to NIPA.^53^ Among them, two lncRNAs, FAM182B and LOC105375785, were found to be involved in the invasiveness of PA and might serve as promising candidate biomarkers for the diagnosis of IPAs.^53^ Another transcriptome sequencing study found 70 differentially expressed lncRNAs between invasive and noninvasive GH‐PAs, wherein epithelial cell differentiation and development pathways were restrained in invasive GH‐PAs by functional annotations and enrichment analysis.^54^ Decreased expression of lncRNA SPINT1‐AS1 was suggested to serve as an indicator of invasive GH‐PAs independent of the unfavorable prognosis.^54^ In addition, the tissue expression of lncRNA ANRIL was increased in patients with IPA compared to that in NIPA patients. In addition, the plasma expression of circulating ANRIL was also increased in patients with IPA before the surgery, however, the plasma circulating ANRIL was significantly decreased in patients with IPA after surgery.^55^ These results indicate that ANRIL might represent a potential biomarker for PA diagnosis.

#### 4.1.2 Prognostic biomarkers

Recurrence is an important factor in refractory PA.^56^ Early prediction of patients at high risk for PA recurrence can help clinicians adjust the treatment plan and thus improve the long‐term cure rate. Therefore, reliable prognostic factors are urgently needed for recurrent PA. Certain clinical prognostic factors (e.g., postoperative residue, age, immunohistological subtypes, invasion, tumor size, hormone levels, and postoperative radiotherapy) and biological prognostic factors (e.g., ki‐67, p53, cadherin, PTTG, MMP‐9, EGFR, fascin actin‐bundling protein 1, cyclooxygenase‐2) have been indicated to predict the risk of recurrence in PA patients.^57^ However, to date, no single factor is adequate to accurately and independently predict PA recurrence.

In recent years, ncRNAs, including lncRNAs, have emerged as novel prognostic biomarkers for predicting the recurrence of PA. For example, lncRNA H19 has been considered as an oncogene in a variety of tumor cells. The expression of plasma exosome‐derived H19 was frequently downregulated in human primary PA and negatively correlated with tumor growth by restraining 4E‐BP1 phosphorylation.^58^ At the same time, H19 can act as a ceRNA to inhibit the expression of miR‐93a and increase the high expression of autophagy related genes (ATG7) in tumor cells, thus enhancing the sensitivity of prolactinoma to dopamine agonists.^34^ Therefore, plasma exosomal H19 may represent a promising prognostic biomarker for medical responses of patients with prolactinoma. Monitoring and predicting the postoperative recurrence of NFPA is usually very difficult, as this PA type is not accompanied by hormone hypersecretion in the serum. A high‐throughput sequencing identified 214 differentially expressed lncRNAs with 120 upregulated and 94 downregulated between fast recurrence NFPA group and slow recurrence NFPA group, among which, several lncRNAs including LL21NC02‐21A1.1 were proved to possess predictive ability for the recurrence of NFPA.^59^ Another study by Kaplan−Meier, random survival forest, and receiver operating characteristic curve analyses identified two lncRNAs COA6‑AS1 and RP11‑23N2.4, which were significantly associated with tumor recurrence.^60^ The combined signature of these two lncRNAs and CHST12 exhibited a high predictive accuracy for tumor regrowth, representing a new marker for predicting the prognosis of patients with NFPA.^60^

### 4.2 LncRNA/miRNA/target protein axis as novel therapeutic target of PA

The ceRNA hypothesis has revealed a new mechanism for the interactions between RNAs.^11^ As a kind of ceRNAs, lncRNAs can competitively bind to miRNAs, which is mediated by miRNA response elements, to regulate gene expression by neutralizing miRNAs' inhibition of target genes.^11^ A recent study reported the construction of a lncRNA‐mediated ceRNA network based on miRNA identification and target prediction, which consisted of 25 lncRNAs, 58 miRNAs, and 67 mRNAs. Among them, KCNQ1OT1, SNHG7, LINC00472, and C9orf72 were thought to function as key molecular sponges by interacting with more than 20 miRNAs and exert important influence in PA development.^61^

Certain differentially expressed lncRNAs are abnormally regulated in IPA tissues and cells, these lncRNAs are shown to participate in cell viability, proliferation, migration, invasion, and EMT by sponging corresponding miRNAs. For example, the SNHG6/miR‐994/RAB11A axis exerted crucial roles in modulating proliferation, migration, invasion, and EMT progress in HP75 cells, indicating SNHG6 and miR‐994 might serve as valuable therapeutic targets for IPA.^62^ The overexpression of lncRNA CYTOR in IPA tissues and cell lines was associated with tumor invasiveness and adenoma size of the patients. Downregulation of CYTOR inhibited PA cell proliferation, migration, and invasion by sponging miR‐206, suggesting the CYTOR‐miR‐206 axis as a tumor‐promoting factor providing new insights into PA treatment.^63^ LncRNA MEG3 was found to inhibit PA progress by negatively modulating miR‐23b‐3p and further rescuing the inhibitory effect of miR‐23b‐3p on FOXO4, indicating the MEG3/miR‐23b‐3p/FOXO4 axis as a potential target for PA treatment.^37^ LncRNA SNHG7 knockdown was shown to delay xenograft tumor progression, and accompanied by upregulated miR‐449a and downregulated Ki67, highlighting the essential oncogenic property of the SNHG7/miR‐449a axis during PA development.^38^ LncRNA PCAT6 was found to modulate the progression of PA by regulating the miR‐139‐3p/BRD4 axis, which might provide a novel biomarker for the targeting treatment of PA.^64^ A recent animal study confirmed that ST8SIA6‐AS1 knockdown blocked the tumor growth of GTI‐1 cells in the nude mouse model with significantly reduced tumor volume and weight, which was possibly mediated via miR‐5195‐3p/HOXA9 axis.^48^ A real‐time PCR analysis confirmed that LINC01116 was abnormally highly expressed in PA cells, its downregulation remarkably inhibited cell proliferation, migration, and EMT progress via the miR‐744‐5p/HOXB8 axis, which could be expected to act as a promising target pathway in PA therapy.^65^ Cumulatively, the interactions between lncRNAs and miRNAs, especially via the ceRNA mechanism, play an important role in PA pathogenesis and can be designed as potential therapeutic targets for PA treatment.

## 5 FUTURE PROSPECTS

Although most PA patients generally have a good prognosis, a certain proportion of them will experience recurrent or progressive PAs characterized by invasion, rapid growth, high recurrence, and poor response to standard treatment. The difficulties that need to be urgently resolved in the diagnosis and treatment of refractory PA include: identifying early biomarkers of refractory PA, determining biomarkers for treatment response, and explicating the combination and sequence of different treatment regimens. The early identification and intervention of refractory PA is difficult and needs to be managed by a multidisciplinary team of experts. For patients suspected of having refractory PA, it is necessary to comprehensively examine and evaluate the imaging, biochemical, and histopathological findings. Currently, temozolomide has been widely used as the first‐line chemotherapy drug for refractory PA; however, its efficacy is usually limited.

Emerging therapeutic strategies are providing alternative options for refractory PA. LncRNAs exert important regulatory functions in the pathogenesis of diverse tumors by promoting cell proliferation, differentiation, migration, invasion, and apoptosis at transcriptional, translational, and posttranslational levels, representing a new class of molecular targets for tumor treatment, including PA. Additionally, circulating lncRNAs could serve as novel biomarkers for early diagnosis and prognosis of PA. Further study of the roles and mechanisms of the reported lncRNAs in PA proliferation and invasion, in particular the mechanisms of lncRNA‐mediated resistance of PA cells to chemotherapy drugs, will shed light on the treatment of refractory PA. In addition, in view of the large number of lncRNAs in humans, and the fact that only a few of them have been functionally studied, it is urgent to develop efficient tools to identify new critical lncRNAs and to explore their roles and mechanisms in the pathogenesis of PA. Furthermore, most of the relevant studies are small case reports, further basic and clinical studies are needed to explore the efficacy and feasibility of novel therapies in refractory PA.

## AUTHOR CONTRIBUTIONS

All authors have written, revised, and approved the manuscript.

## CONFLICT OF INTEREST STATEMENT

The authors declare no conflict of interest.

## Data Availability

Data are available upon reasonable request.
